# Disclosure of Alzheimer’s Disease Diagnosis: Patient Preferences and Physician Approach

**DOI:** 10.1002/alz.093589

**Published:** 2025-01-09

**Authors:** Maria Elvira Söderlund, Maria Elena Guajardo, Marina Cavagna, Edith Labos, Marcelo Schapira, Daniel Bernardo Seinhart

**Affiliations:** ^1^ Hospital Italiano de Buenos Aires, Buenos Aires Argentina

## Abstract

**Introduction:**

The announcement of an Alzheimer’s disease (AD) diagnosis is a complex process that requires careful consideration by healthcare professionals. This study aims to explore the desires of patients regarding the disclosure of their Alzheimer’s diagnosis and to understand the practices adopted by physicians in this regard.

**Methods:**

Within the context of memory disorders consultations, patients were queried about their preferences regarding the disclosure of an AD diagnosis if it were determined to be the cause of their memory issues. Additionally, a survey was conducted among physicians specializing in the care of older adults, specifically at the Italian Hospital of Buenos Aires, to assess their practices and concerns related to the disclosure of AD diagnoses.

**Results:**

Out of 250 patients, 223 expressed a desire to know their diagnosis, while 19 were uncertain, and 7 declined. Among the 76 participating physicians, the majority (82.9%) were aged between 35 and 65 years, with 68% being female. A significant portion (58.7%) were geriatricians while 27.8% were primary care physicians. Notably, 32% of physicians reported hardly ever using AD when delivering a diagnosis and 48% preferring the term “cognitive impairment”. Only 2 physicians always use AD in this context. The main reasons for avoiding this term were the potential risk of psychological impact (43.8%) and stigmatization (20.5%) associated with the diagnosis.

**Conclusions:**

The incorporation of biomarkers enhances diagnostic certainty and therapeutic possibilities for AD. While most patients express a desire to know their diagnosis, a significant proportion of physicians opt for more general terms such as “cognitive impairment” instead of explicitly using the term AD. Understanding patient preferences and providing information in a sensitive manner are crucial aspects in the disclosure process, aiming to minimize potential harm. The right of patients to know or not to know their diagnosis should be respected, highlighting the importance of ethical and patient‐centered approaches in AD diagnosis disclosure.

